# Stereotypical Questions: How Stereotypes About Conversation Partners Are Reflected in Question Formulations

**DOI:** 10.1177/01461672231205084

**Published:** 2023-10-21

**Authors:** Camiel J. Beukeboom, Christian Burgers, Maxim van Woerkom, Sibren de Meijer, Laura de Vries, Denise Ferdinandus

**Affiliations:** 1 Vrije Universiteit Amsterdam, The Netherlands; 2 University of Amsterdam, The Netherlands

**Keywords:** stereotypes, questions, linguistic bias, confirmation bias, interpersonal communication

## Abstract

In conversations, activated stereotypes about conversation partners can influence communicative behaviors. We investigate whether and how stereotypes about categorized conversation partners shape topic choice and the types of questions asked. In three experiments, participants imagined having a conversation. Gender or age stereotypes of the conversation partner were manipulated by means of a picture. Results show a higher likelihood of addressing conversation and question topics consistent with stereotypic expectancies about conversation partners. Moreover, stereotypes were reflected in subtle variations in question formulations. When questions address stereotype-consistent topics, they are likelier formulated with high-frequency adverbs and positive valence, while questions addressing stereotype-inconsistent topics more likely contain low-frequency adverbs and negative valence. In addition, Experiment 4 suggests that recipients are sensitive to detect that questions reflect stereotypes about themselves, which can influence the evaluation of the conversation and partner. We discuss the consequences of biased question asking for interpersonal conversation and stereotype maintenance.

Anecdotal evidence suggests that mothers are asked more questions about how they manage to balance their work and childcare than fathers (e.g., Reimer, 2020; Ryder, 2018). Apparently, stereotypic expectancies of the questioner (e.g., the expectancy that raising children is a woman’s task) are reflected in the questions asked. Because questions strongly determine the subsequent course and content of conversations, biases in questions have the potential to reinforce stereotypes. It is, therefore, important to understand how stereotypes are reflected in language use, such as questions. Indeed, research on biased language use reveals that our language echoes stereotypic expectancies about categorized individuals when we communicate *about* other people and their behavior (Beukeboom & Burgers, 2019). However, little research has been conducted on how stereotypic expectancies influence our communication *with* categorized individuals, such as the type of questions that are asked to categorized conversation partners (Beukeboom & Burgers, 2017).

When interacting with others, people draw various inferences about their conversation partner’s thoughts, goals, intentions, and attitudes. Such inferences are crucial in the coordination of the conversation and are needed to obtain mutual understanding (Krauss & Fussell, 1996). Importantly, inferences drawn are often (partly) based on activated stereotypes applied to the conversation partner. Research in the area of communication accommodation theory (Dragojevic et al., 2015) and interracial (Trawalter et al., 2009) and intergenerational (Hummert et al., 2004) communication, for instance, shows how activated stereotypes about conversation partners influence communicative behaviors. A relatively unexplored question is whether and how activated stereotypes determine the types of questions asked.

The relationship between stereotype activation and question asking is important, because the specific questions asked typically influence answers given by recipients, and thereby strongly impact the course, content, and outcome of the conversation (see Loftus, 1975; Semin & De Poot, 1997a, 1997b). Specifically, questions have been shown to play a role in a confirmatory hypothesis testing process (Fiedler et al., 1999; Klayman & Ha, 1987; Snyder et al., 1977). That is, a tendency to rely on positive hypothesis testing (i.e., one-sided information seeking) on one hand, and a tendency of recipients to acquiesce in providing confirmatory (rather than contradicting) responses on the other hand is often argued to result in hypothesis confirmation (Fiedler & Walther, 2003; Holtgraves, 2001; Zuckerman et al., 1995). Although much research has addressed this confirmatory hypothesis testing process, relatively little is known about the linguistic elements of the first part, that is, how hypotheses—and specifically stereotype-induced hypotheses—guide question formulation.

In the present article, we study whether and how stereotypes about categorized conversation partners shape conversation topics and the content and specific formulations of questions asked. In addition, we explore the potential consequences of biased question asking in addressees and link to the role of stigma consciousness. We then discuss how biased question asking could (inadvertently) result in stereotype confirmation about the target (Dumont et al., 2003; Snyder & Stukas, 1999), and thereby play a role in stereotype maintenance.

## Stereotypes and Question Asking

When interacting with others, people adapt their communication to their conversation partners. We may simplify an explanation when talking to a child or talk louder when speaking with someone with hearing difficulties. In doing so, we may rely on stereotypic expectancies. Socially categorizing a conversation partner allows us to quickly and efficiently draw stereotypic impressions which can guide our communicative behaviors, particularly when interacting with people we have just met (Macrae & Bodenhausen, 2000). In many cases, however, relying on generalized (negative) stereotypic beliefs may lead us astray, because we are interacting with individuals with idiosyncratic characteristics and needs. In addition, relying on stereotypes may be demeaning to the other. Research on intergenerational communication, for instance, shows that younger adults, when relying on negative age stereotypes, tend to over adjust their communication when communicating with elderly people, for example, by using simplified grammar and vocabulary, unnecessary repetition, slow speech rate, and exaggerated intonation (Dragojevic et al., 2015; Hummert et al., 2004).

Social categorization and stereotypes thus play a role in interactions, but it remains unclear how activated stereotypes about conversation partners influence the type of questions asked. Particularly relevant for this issue is research on (social) information-seeking behavior and people’s tendency to engage in positive hypothesis testing (Klayman & Ha, 1987). The positive test strategy implies that people tend to test hypotheses by examining instances in which the property or event is expected to occur (to see whether it does occur; Klayman & Ha, 1987). Numerous studies have indeed shown that people are likelier to ask questions about features in line with their prior hypotheses rather than with alternative hypotheses (for reviews, see Nickerson, 1998; Sanbonmatsu et al., 1998; Trope & Liberman, 1996). In other words, people prefer asking questions to which they expect an affirmative (yes) answer.

In a classic demonstration of this effect, Wason (1960) asked participants to find the rule according to which triples of numbers (e.g., 2–4–6) were constructed. Participants could generate their own triples and ask the experimenter whether or not it conformed to the rule. Participants overwhelmingly tested triples that obeyed their initially hypothesized rule. For example, when they hypothesized that the correct rule was “three consecutive even numbers,” they asked about triples that obeyed their hypothesized rule (e.g., 6–8–10) rather than triples that violate this rule such as (3–5–7).

In a comparable study (Snyder & Swann, 1978), participants tested whether someone was either extraverted or introverted by selecting 12 from a list of 26 questions. Eleven of these questions would be asked of people known to be extraverts (extraversion-biased questions; e.g., What would you do if you wanted to liven things up at a party?), while 10 other questions would be asked of people known to be introverts (introversion-biased questions; e.g., In what situations do you wish you could be more outgoing?); the remaining questions were neutral. Participants asked to test whether someone was extraverted mostly selected extraversion-biased questions, whereas participants asked to test whether someone was introverted mostly selected introversion-biased questions. Thus, people preferentially ask questions that match their hypotheses, and to which they expect affirmative answers. Subsequent work confirmed this tendency when questions were freely formulated, and showed that the tendency is stronger when participants were more certain of their beliefs (Swann & Giuliano, 1987).

Because activated stereotypes bring immediate expectancies and hypotheses about a conversation partner’s interests and activities, we predict that stereotypes shape questions in a similar way. Yet, despite great interest in positive hypothesis testing and confirmation bias, only a few studies have focused on the role of stereotypes in social information seeking and/or whether this induces stereotype confirmation (Cameron & Trope, 2004; Fiedler et al., 1999; Sacchi et al., 2012; Trope & Thompson, 1997). Of particular interest for our focus are the few relevant studies that specifically focused on the role of activated stereotypes in question-asking behavior (Dumont et al., 2003; Johnston, 1996).

In the work by Johnston (1996, Experiments 1 and 3), participants gathered information by selecting topics and questions in a bulletin board about four exemplar members of the target category “doctors.” Participants could gather information by first selecting a topic to ask a question about. These topics focused on stereotype-consistent, stereotype-inconsistent, or stereotype-neutral traits. Next, participants selected one of the two questions on this topic, which was also either stereotype-consistent or stereotype-inconsistent. First, results revealed a stereotype preservation bias, in that participants showed a bias toward selecting stereotype-consistent topics. However, there was no evidence of a positive test strategy in question choice, because subjects selected equal proportions of stereotype-consistent and stereotype-inconsistent questions. The latter finding may be due to the fact that the questions used in the experimental materials varied widely in content and formulation.

In a comparable study by Dumont et al. (2003), participants rated the extent to which they wanted to ask a number of questions to a person presented as a female hairdresser. Given that hairdressers were stereotypically perceived as sociable but not very intelligent, a positive test strategy was expected in a preference for questions testing for lack of intelligence (e.g., “Are you interested in the success of [the TV show] Top Models?”) or presence of sociability (e.g., “Do you often go out with your friends?”). The pattern of results was not fully in line with this hypothesis, which may be partly due to low statistical power (*n* = 11–15 per experimental condition).

Although these studies (Dumont et al., 2003; Johnston, 1996) suggest that activated stereotypes about a conversation influence topic choice, they do not provide clear evidence about whether and how stereotypes influence question asking. They also do not address specific differences in question formulation, which was also not addressed in earlier positive hypothesis testing research. Note that the biased questions that participants could select varied in unsystematic ways. For instance, some expressed an expected positive or negative preference (e.g., “Are you interested in the success of Top Models?”, Dumont et al., 2003); “What do you dislike about parties?” Snyder & Swann, 1978), some presented an expected trait in question content (e.g., “In what situations are you most talkative?”, Snyder & Swann, 1978), while other questions express an expected frequency of activity (e.g., “Do you *often* go out with your friends?”, Dumont et al., 2003). Research on question wording (Semin & De Poot, 1997a, 1997b), however, has shown that very subtle variations in formulation (e.g., verb types) may both result from expectations of the questioner and affect recipients. Another limitation is that the experimental tasks are not necessarily ecologically valid. In real life, we rarely “test” for a personality trait (Snyder & Swann, 1978), and the expectancies that may guide our interactive behavior are not made explicit but instead follow from unconsciously activated stereotypes.

We think it is important to gain a better understanding on how stereotypes influence question asking, because biased questions may eventually lead to stereotype confirmation about the target (Dumont et al., 2003; Fiedler & Walther, 2003; Snyder & Stukas, 1999), and thus play a role in stereotype maintenance. Several reasons have been put forward for the idea that positive testing leads to the probable confirmation of the tested hypothesis, even if it is not true. First, biased questions are often leading and thereby invite a confirmative answer. In Snyder and Swann’s (1978) study, for instance, people testing if someone is extraverted may ask what that person would do to liven up a party. Considering that most people, including introverted ones, would be able to answer this question, this type of question tends to lead to a confirmation of the hypothesis tested (cf. Snyder, 1992). Second, recipients tend to acquiesce, which means that they are likelier to agree than disagree and to go along with the direction initiated by one’s conversation partner (Holtgraves, 2001; Zuckerman et al., 1995). Positive hypothesis testing thus tends to result in a relatively higher amount of (confirmatory) learning input that matches the given hypothesis, and this alone can result in its confirmation (Fiedler & Walther, 2003; Fiedler et al., 1999). Moreover, even if responses are disconfirming, questioners have a tendency toward interpreting answers in a stereotype-confirming manner (Cameron & Trope, 2004).

Thus, the tendency for positive hypothesis testing appears to be a robust phenomenon and to contribute to confirmation of one’s hypothesis, although there still is discussion on the underlying mechanisms and important boundary conditions have been identified (e.g., Evans, 1998; McKenzie, 2004; Swann & Giuliano, 1987; Trope & Liberman, 1996). Still, not much work focused specifically on how stereotypes about a conversation partner affect question asking, and particularly how stereotypes result in specific variations in question formulation remains unknown.

## The Present Research

In these studies, we aim to unravel whether stereotypes result in positive hypothesis testing and how this is expressed in topic choice and question formulation. We test whether and how stereotypes associated with a categorized conversation partner shape questions asked in an imagined conversation (Experiments 1–3), and, in addition, explore how stereotypically biased questions are perceived by recipients (Experiment 4). In contrast to work by Johnston (1996) and Dumont et al., 2003, we unobtrusively vary the conversation partner’s social category (and thereby the associated stereotype) merely by means of a photograph. In doing so, we contrast two opposing categories from the same domain (i.e., age stereotypes: old vs. young in Experiments 1 and 3; gender stereotypes: male vs. female in Experiment 2). Second, we specifically focus on different variations in question formulation and explore how speakers’ stereotypic expectancies about conversation partners may be reflected in the questions asked.

First, in line with Johnston (1996) and Dumont et al. (2003), we expect that stereotypes determine conversation topic choice. Following the literature on positive hypothesis testing (Nickerson, 1998), we predict that people are likelier to address topics that are stereotype-consistent (vs. stereotype-inconsistent) with the conversation partner’s social category. This can both be explained from an intrapersonal social cognition perspective as well as from an interpersonal pragmatics, and social relationship perspective. That is, research on stereotyping shows that people automatically activate the associated mental representations once they socially categorize a person (Macrae & Bodenhausen, 2000). This means that stereotype-consistent information is cognitively activated and thus likelier expressed in language use, while stereotype-inconsistent information is inhibited (Wigboldus et al., 2003). Next, it can be expected that people are likeliest to introduce relevant topics which they expect to resonate with their conversation partner (Grice, 1975). Finally, introducing stereotype-consistent (vs. stereotype-inconsistent) topics and questions can serve to maintain a more pleasant interaction and social connection with a conversation partner (Clark & Kashima, 2007). This leads to

**Hypothesis 1 (H1):** In conversations with unfamiliar conversation partners, speakers are likelier to (a) introduce conversation topics and (b) prefer question topics that are stereotype-consistent (vs. stereotype-inconsistent) with the social category of their conversation partner.

Once a conversation topic has been chosen, we expect that question formulation may also reveal the speaker’s stereotypic expectancies in more subtle ways. One likely stereotype-induced formulation variation is the use of frequency adverbs (e.g., frequently, occasionally). Research by Loftus (1975) suggests that frequency adverbs in questions (e.g., Do you get headaches . . . “frequently” or “occasionally?”) have the potential to communicate presuppositions, which are picked up by respondents. We hypothesize that when questioners formulate questions about stereotype-consistent activities for their conversation partner, a relatively high frequency of such activities is expected and expressed in their questions (Do you *often* go out with friends). When the same activities are stereotypically unexpected for their conversation partner, we expect this unexpectedness to be expressed in a likelier use of low-frequency adverbs (e.g., Do you *sometimes* go out with friends?). This leads to

**Hypothesis 2a (H2a):** When questions address stereotype-consistent (vs. stereotype-inconsistent) activities, people prefer questions with high-frequency (vs. low-frequency) adverbs.
**Hypothesis 2b (H2b):** When questions address stereotype-inconsistent (vs. stereotype-consistent) activities, people prefer questions with low-frequency (vs. high-frequency) adverbs.

We report all materials, manipulations, measures, exclusions, power analyses, additional results, and data and syntax of all experiments in online appendices: https://osf.io/jc3fr/. Studies were not preregistered.

## Experiment 1

### Method

#### Participants and Design

Participants were recruited via snowball sampling using different social media (e.g., Facebook and LinkedIn). This resulted in a convenience sample of 135 Dutch voluntary participants who completed an online experimental questionnaire; 40 males (30%), 95 females (70%), *M*_age_ = 27.9 years, *SD* = 10.5, minimum = 17, maximum = 67; 1 missing value.

Participants were told that the study focused on the course of getting-acquainted conversations and asking questions, and then imagined having an informal conversation with somebody they did not know. In this between-subjects experiment, participants were randomly presented with a photograph of either a young or old male conversation partner. Subsequently, participants rated conversation topics, and various potential questions for their conversation partners which varied in topic (stereotype-consistent vs. stereotype-inconsistent) and formulation (high-frequency vs. low-frequency adverbs).

#### Materials and Procedure

To manipulate conversation partner age, we obtained emotionally neutral photographs (young and old) from the Aging Mind Face Database (Minear & Park, 2004). In the condition with a young conversation partner, participants were randomly presented with one of the three photographs of young males between 18 and 23 years. In the condition with an old conversation partner, participants saw one of the three photographs of older males (>75 years; see Appendix Table A1). This photograph was only shown on a first instruction page and was presented as an unimportant additional feature (i.e., to give you a better impression of the person you are having a getting-acquainted conversation with) to keep participants unaware that we were interested in age or stereotypes.

For our operationalization of the dependent variables, we conducted a small pilot study (*N* = 16) to obtain activities that were perceived as either stereotypically young, stereotypically old, or stereotypically neutral (see Appendix Table A3 for details). As a manipulation check, participants in the main study rated expectedness of these activities for young and old persons on 7-point Likert-type scales (1 = *not at all expected*; 7 = *very much expected*), but only after measuring the dependent variables to prevent potential demand effects.

As dependent variable, participants rated the *likelihood of conversation topic choice* by indicating the likelihood of asking the person in the photograph about this topic (1 = *not at all likely*; 7 = *very likely*). We recoded these variables such that they indicated the likelihood of choosing stereotype-consistent and stereotype-inconsistent topics.

The same pretested activities were used to create stereotype-consistent and stereotype-inconsistent questions with variations in formulation in (a) addressed topic and (b) frequency adverbs. Participants were presented with 13 pairs of questions that were similar in focus, but with small variations in content or frequency adverbs (to conceal the manipulation, three pairs were neutral fillers). For each question, participants indicated the likelihood of asking it to their conversation partner (1 = *not at all likely*; 7 = *very likely*).

To measure *preferred question topic*, we presented four question pairs with questions varying in the topics. In each pair, one question addressed a stereotypically young-age activity and the other question addressed a stereotypically old-age activity. Two question pairs asked whether the addressee engaged in a specific activity and two asked about the addressee’s preference for the activity. Questions in each pair varied in only one word referring to the activity (e.g., *Do you run/walk to stay fit?*). Questions were recoded depending on conversation partner age condition, such that they indicated the *preference for stereotype-consistent and stereotype-inconsistent question topics*.

To measure *preferred question frequency*, we created six question pairs varying in using either a high-frequency adverb (e.g., often) or a low-frequency adverb (e.g., sometimes). Three pairs addressed stereotypically young activities (e.g., *Do you often/sometimes listen to pop music?*), and three pairs addressed stereotypically old activities (e.g., *Do you often/sometimes listen to classical music?*). Next, we calculated mean ratings for high-frequency questions addressing stereotypically young or old activities, and mean ratings for low-frequency questions addressing stereotypically young or old activities. Again, variables were recoded to whether they were stereotype-consistent or stereotype-inconsistent with the conversation partner.

### Results

#### Manipulation Check

A randomization check showed that participants were equally distributed across the six conversation partner photograph conditions (young conversation partner: Photograph 1: *n* = 22, Photograph 2: *n* = 19, and Photograph 3: *n* = 26; old conversation partner: Photograph 1: *n* = 23, Photograph 2: *n* = 24, and Photograph 3: *n* = 21). These were collapsed to create the two conversation partner age (young and old) conditions (*n* = 67 and *n* = 68, respectively). We observed no significant differences between the six photograph conditions or the collapsed conversation partner age (young and old) conditions in participant age or gender (*F*s < 1).^1^

In line with the pilot study, the manipulation check showed that stereotypically young-age activities were significantly more expected for young (*M* = 5.98, *SD* = 0.65) than for old people (*M* = 2.10, *SD* = 0.71), *t*(134) = 40.77, *p* < .001, *d* = 3.51. Similarly, stereotypically old-age activities were more expected for old (*M* = 5.54, *SD* = 0.63) than young people (*M* = 3.02, *SD* = 0.80), *t*(134) = 29.90, *p* < .001, *d* = 2.57.

#### Likelihood of Conversation Topic Choice

*H1a* predicted that people are likelier to address stereotype-consistent (vs. stereotype-inconsistent) topics. To test this hypothesis, we conducted a 2 (conversation partner age: young and old) × 2 (topics addressing stereotype-consistent vs. stereotype-inconsistent activities) mixed analysis of variance (MANOVA), with repeated measures on the last factor. Analyses that did not directly test one of the hypotheses are reported in the Digital Appendix.

Confirming *H1a*, participants indicated a higher likelihood of addressing stereotype-consistent (vs. stereotype-inconsistent) topics, *F*(1,133) = 534.71, *p* < .001, *ηp*^2^ = .80; see Table 1 for descriptive statistics. While we observed no main effect of conversation partner age, *F*(1,133) = 2.47, *p* = .12, *η_p_*^2^ = .02, we did find an interaction effect showing that the above main effect of likelihood of topic choice was somewhat stronger in the old (vs. young) conversation partner condition (see Table A4).

**Table 1.** Means (and SD) of Likelihood of Conversation Topic Choice, Preferred Question Topic, Valence, and Frequency in Questions, of Topics and Questions Addressing Age or Gender Stereotype-Consistent, Stereotype-Inconsistent, and Stereotype-Neutral Activities (Experiments 1 and 2).

	Stereotype consistency of addressed activity		
	Consistent	Inconsistent	Neutral
**Experiment 1 (age stereotypes)**			
Likelihood conversation topic choice	4.50^a^ (1.11)	2.11^b^ (0.75)	—
Preferred question topic	4.32^a^ (1.29)	2.49^b^ (1.10)	—
Preferred frequency in questions			
High frequency	3.99^a^ (1.48)	2.17^b^ (1.09)	—
Low frequency	4.71^a^ (1.25)	3.52^b^ (1.30)	—
**Experiment 2 (gender stereotypes)**			
Likelihood conversation topic choice	3.89^a^ (1.43)	2.14^b^ (0.97)	3.89^a^ (1.10)
Preferred question valence			
Positive valence	4.26^a^ (1.31)	3.05^b^ (1.34)	4.40^a^ (1.17)
Negative valence	2.60^a^ (1.31)	2.94^b^ (1.45)	2.35^c^ (1.14)
Preferred frequency in questions			
High frequency	3.70^a^ (1.44)	2.49^b^ (1.21)	3.54^a^ (1.10)
Low frequency	3.99^a^ (1.22)	3.19^b^ (1.23)	4.07^a^ (1.09)

*Note*. Experiment 1: *N* = 135 and Experiment 2: *N* = 236. Means in rows with different subscript (^a, b, c^) are significantly different (*p* < .01) according to Bonferroni-adjusted pairwise comparisons. Ratings indicate how likely respondents would choose topic/ask question to their conversation partner (1 = very unlikely; 7 = very likely).

#### Preferred Question Topic

*H1b* predicted that speakers would prefer questions addressing stereotype-consistent (vs. stereotype-inconsistent) topics. To test *H1b*, we conducted a similar MANOVA as for *H1a*, but with preferred question topic (stereotype-consistent vs. stereotype-inconsistent) as the repeated-measures factor.

Confirming *H1b*, participants indicated a higher preference for questions addressing stereotype-consistent (vs. stereotype-inconsistent) topics, *F*(1,133) = 132.37, *p* < .001, *η_p_*^2^ = .50. We also found that participants in the old (vs. young) conversation partner condition had a higher overall preference for questions on any topic, *F*(1,133) = 5.35, *p* = .02, *η_p_*^2^ = .04. The interaction of preferred question topic and conversation partner age was not significant, *F* < 1.

#### Preferred Frequency in Question Formulation

*H2* predicted that people would prefer questions with high-frequency adverbs when the question addressed stereotype-consistent (vs. stereotype-inconsistent) activities (*H2a*), while people would prefer questions with low-frequency adverbs when the question addressed stereotype-inconsistent (vs. stereotype-consistent) activities (*H2b*). To test *H2*, we conducted a 2 (conversation partner age: young vs. old) × 2 (high-frequency vs. low-frequency adverbs) × 2 (questions with stereotype-consistent vs. stereotype-inconsistent activities) MANOVA, with repeated measures on the last two factors.

First, we found that, across different frequency formulations, participants preferred stereotype-consistent (vs. stereotype-inconsistent) questions, *F*(1,133) = 135.53, *p* < .001, *η_p_*^2^ = .51, again confirming *H1b*. More importantly for *H2*, we found an interaction between preferred frequency and question topic, *F*(1,133) = 14.03, *p* < .001, *η_p_*^2^ = .10. This interaction shows that the main effect of question topic (stereotype-consistent vs. stereotype-inconsistent) is qualified by the type of frequency adverb used in the question. Confirming *H2a*, participants indicated a stronger preference for high-frequency questions when these address stereotype-consistent (vs. stereotype-inconsistent) activities. The pattern for low-frequency questions is at first sight not in line with *H2b* as participants still indicated a higher preference for low-frequency questions addressing stereotype-consistent over stereotype-inconsistent activities (which disconfirms *H2b*), but the two main effects obscure the pattern. Table 1 and mean comparisons (see Online Appendix) show that in questions addressing stereotype-inconsistent topics, the preference for low-frequency formulations is enhanced, while the preference for high-frequency formulations declines. In questions addressing stereotype-consistent topics, in contrast, the preference for high-frequency (vs. low-frequency) formulations is enhanced. Together, this confirms *H2*.

## Experiment 2

Experiment 1 confirmed that age stereotypes associated with a conversation partner impact the choice of conversation topics and question formulations. To test the robustness and generalizability of these findings, we conducted Experiment 2. This is a conceptual replication of Experiment 1 with a few changes. First, Experiment 2 focused on gender (rather than age) stereotypes. Second, in testing preferences for question formulations, we added another variation focusing on question valence. We vary in formulations indicating a positive versus a negative preference of stereotypically male and female activities (e.g., *Do you think gaming is fun/boring?*). We expect that stereotypes induce an expected positive or negative preference for an addressed activity, which can be conveyed in question valence. Accordingly, we hypothesize that participants will show preferences for formulations in line with stereotypic expectancies, leading to

**Hypothesis 3a (H3a):** When questions address stereotype-consistent (vs. stereotype-inconsistent) activities, speakers prefer questions with positive (vs. negative) valence.
**Hypothesis 3b (H3b):** When questions address stereotype-inconsistent (vs. stereotype-consistent) activities, speakers prefer questions with negative (vs. positive) valence.

### Method

#### Participants and Design

Participants were recruited via snowball sampling using different social media (e.g., *Facebook and LinkedIn*). This resulted in a convenience sample of 236 Dutch voluntary participants who completed the online experimental questionnaire, 100 males (42%), 136 females (58%), *M*_age_ = 27.7 years, *SD* = 10.1, minimum = 16, maximum = 68. We excluded cases below 16 years of age (*n* = 2).

The set-up was similar to Experiment 1, but participants were now randomly presented with photographs of male or female conversation partners. We also added a measurement of topics and questions about stereotype-neutral activities as a baseline comparison, and participants, in addition, rated questions that varied in formulation valence: positive and negative.

#### Materials and Procedure

Experimental procedure and instructions were equal to Experiment 1. To vary conversation partner gender, we obtained six emotionally neutral conversation partner photographs (three men and three women, all in their early 20s) from the Aging Mind Face Database (Minear & Park, 2004; see Appendix Table A5). In contrast to Experiment 1, the conversation partner photograph remained visible on the screens measuring the dependent variables. Similar to Experiment 1, we conducted a small pilot study to obtain activities that were perceived as either stereotypically male, stereotypically female, or gender stereotype neutral (see Appendix Table A6 for details). We used the same measure as in Experiment 1 to measure *likelihood of conversation topic choice.*

Next, we presented participants with 24 question pairs varying in (a) frequency and (b) valence. For both frequency and valence, we had four question pairs each to address stereotypically male, female, and neutral activities. *Preferred question frequency* was measured similarly to Experiment 1 for questions with high-frequency and low-frequency adverbs.

To measure *preferred question valence*, questions in each pair varied only in one word stating either a positive valence (e.g., *fun*) or a negative valence (e.g., *boring*) toward the addressed activity. Preferred question valence was subsequently calculated for positive and negative questions addressing stereotypically male, female, and neutral activities separately.

Like in Experiment 1, we recoded the dependent variables (except the neutral ones) to reflect the preference for stereotype-consistent and stereotype-inconsistent topics and questions with respect to conversation partner sex (see Appendix Table A7 for all questions).

### Results

#### Manipulation Check

A randomization check showed that participants were equally distributed across the six conversation partner photograph conditions (male: Photograph 1: *n* = 42, Photograph 2: *n* = 36, and Photograph 3: *n* = 43; female: Photograph 1: *n* = 40, Photograph 2: *n* = 40, and Photograph 3: *n* = 35). These were collapsed to create the two conversation partner gender (male and female) conditions (*n* = 121 and *n* = 115, respectively). No differences were observed between the collapsed conversation partner gender conditions in participant age or gender, *F* < 1.^1^

In line with the pilot study, the manipulation check showed that stereotypically male activities were significantly more expected for men (*M* = 5.89, *SD* = 0.75) than for women (*M* = 3.30, *SD* = 0.84), *t*(235) = 31.77, *p* < .001, *d* = 2.07, and that stereotypically female activities were more expected for women (*M* = 5.84, *SD* = 0.76) than for men (*M* = 2.87, *SD* = 0.73), *t*(235) = 37.65, *p* < .001, *d* = 2.45. Stereotypically neutral activities were perceived as slightly more expected for men (*M* = 4.79, *SD* = 0.85) than for women (*M* = 4.69, *SD* = 0.86), *t*(235) = 3.53, *p* = .001, *d* = 0.23.

#### Hypothesis Testing

Hypotheses were tested in a similar way to Experiment 1. Confirming *H1a*, participants indicated a lower likelihood of addressing stereotype-inconsistent topics rather than both stereotype-consistent and stereotype-neutral topics, *F*(2,486) = 242.54, *p* < .001, *η_p_*^2^ = .51 (see Table 1 for descriptive statistics).

Next, we focused on question formulations using different frequency adverbs across question topics. First, in line with Experiment 1 and *H1b*, participants had a higher preference for questions with stereotype-consistent or stereotype-neutral content compared with stereotype-inconsistent content. We also again observed a main effect of preferred frequency in questions, showing that low-frequency questions and neutral ones were overall more preferred than high-frequency questions.

Next, and most relevant for *H2*, we again observed an interaction between preferred frequency and question topic, *F*(2,468) = 11.68, *p* < .001, *η_p_*^2^ = .05, showing that the main effect of question topic was qualified by the type of frequency adverb used in the question. Like in Experiment 1, participants indicate a much stronger preference for high-frequency questions addressing stereotype-consistent (vs. stereotype-inconsistent) topics (confirming *H2a*). For low-frequency questions, the pattern is more complex, but in line with Experiment 1. Table 1 and mean comparisons (see Online Appendix) show that in questions addressing gender stereotype-inconsistent topics, the preference for low-frequency formulations is enhanced, while the preference for high-frequency formulations declines. In questions addressing stereotype-consistent topics, in contrast, the preference for high-frequency (vs. low-frequency) formulations is enhanced. Together, these results replicate Experiment 1 and generally confirm *H2*.

Finally, we tested *H3*, which predicted that speakers would prefer positively valenced questions for stereotype-consistent (vs. stereotype-inconsistent) activities (*H3a*), and that speakers would prefer negatively valenced questions for stereotype-inconsistent (vs. stereotype-consistent) activities (*H3b*). In line with Experiment 1 and *H1b*, we found that people preferred questions addressing stereotype-consistent topics and stereotype-neutral topics compared with stereotype-inconsistent topics, *F*(2,468) = 44.01, *p* < .001, *η_p_*^2^ = .16. Most relevant for *H3*, we observed an interaction between preferred question valence and question topic, *F*(2,468) = 123.99, *p* < .001, *η_p_*^2^ = 35, showing that the main effect of question topic (stereotype-consistent over stereotype-inconsistent) was qualified by question valence. Participants indicate a stronger preference for positively formulated questions when these addressed stereotype-consistent compared with stereotype-inconsistent activities. For negatively formulated questions, however, this is reversed: Participant indicates a stronger preference for negatively formulated questions when these address stereotype-inconsistent compared with stereotype-consistent activities (see Table 1). Together, this confirms *H3*.

## Experiment 3

Experiments 1 and 2 confirmed our hypotheses about adapting topic choice and question formulation to conversation partner age and gender stereotypes. In Experiment 3, we aimed to further extend this evidence by again presenting participants with a conversation partner (young vs. old), but now asked them to spontaneously come up with potential conversation topics, and to formulate their own questions.

### Method

#### Participants and Design

Participants were recruited via snowball sampling using different social media (e.g., *Facebook and LinkedIn*). This resulted in a convenience sample of *N* = 153 Dutch voluntary participants who completed the online experimental questionnaire, 49 males (32%), 103 females (67%), and 1 missing; *M*_age_ 30.8 years, *SD* = 12.6, minimum = 19, maximum = 65. Three cases less than 16 years (*n* = 3) were excluded.

The design and manipulation of conversation partner age (young vs. old) was similar to Experiment 1. Participants now came up with their own conversation topics, and they formulated their own questions to address prompted topics about stereotypically young-age activities, stereotypically old-age activities, stereotypically neutral activities. Respondents then rated their self-generated topics and questions on stereotypicality. In addition, the self-generated questions were coded for the use of valence words (positive and negative) and frequency adverbs (high and low).

#### Materials and Procedure

The scenario with the introduction of the conversation partner was comparable to Experiments 1 and 2. In our manipulation of conversation partner age, we used a different set of six photographs with old and young conversation partners to demonstrate generalizability. Furthermore, after the introduction to the conversation partner, participants were now asked to spontaneously generate conversation topics they would like to talk about to get to know the person. They could indicate up to five conversation topics in separate text boxes.

Next, participants were presented with 13 conversation topics addressing different activities (each on a separate page) and, for each topic, asked to spontaneously formulate up to four questions in open text boxes. Instructions read “The conversation now turns to [e.g., music]. You are talking about [*rap music*]. What question or questions would you ask your conversation partner about this topic?”. Five conversation topics addressed stereotypically young-age activities, five addressed stereotypically old-age activities, and three addressed stereotypically neutral activities. Topics were based on the materials of Experiment 1. While each topic was related to a broad category (e.g., music), the specific topic activity was stereotypically associated with young people, old people, or stereotype-neutral (e.g., rap music, classical music, and Sky Radio). The order of the topics was randomized for each participant and always started with a neutral topic.

Next, participants were presented with the literal conversation topics they previously entered, and *rated conversation topic stereotypicality* by indicating the extent to which they would typically discuss each topic with a young or old person on scales ranging from 1 = typical for a young person to 7 = typical for an old person. The variable consists of the mean rating across the entered topics (up to five). We also computed a variable for *number of entered topics.*

Subsequently, participants were presented with the literal questions they had previously entered. All questions they had formulated (up to four for each of the 13 activities) were rated on two scales. To measure *expected question affirmation*, participants indicated the extent to which they expected either a negative denying answer (no) or a positive affirmative answer (yes) to the question on scales ranging from 1 = *probably a negative answer* to 7 = *probably a positive answer*. To measure *rated question stereotypicality*, participants assessed the extent to which they would typically ask each question to a young or old person on the same 7-point scale as above. For this variable, we computed three scores consisting of the mean ratings across the formulated questions for the stereotypically young, stereotypically old, and stereotypically neutral activities. We also created a variable for *mean number of entered questions* for stereotypically young, stereotypically old, and stereotypically neutral activities (score range: 0–4).^2^

As a manipulation check of the conversation topics, we next measured the perceived *activity stereotypicality* of the 13 activities about which participants formulated their questions, on two scales ranging from 1 = *not at all expected for youngsters/elderly* to 7 = *very much expected for youngsters/elderly*. Based on these, we computed the average rating for stereotypically old, stereotypically young, and stereotypically neutral activities.

As a *manipulation check of conversation partner age* participants were asked to which extent they perceived their conversation as belonging to one of the groups (1 = *youngsters*; 7 = *elderly*), and they estimated the age of their conversation partner.

#### Coding Question Formulations

The questions formulated by participants were coded for the use of *valence words* (positive and negative) and *frequency adverbs* (high-frequency and low-frequency) referring to the addressed activity in the question. For these variables, we computed the proportion of questions addressing stereotypically young/old/neutral activities that contained positive and negative valence words, and high- and low-frequency adverbs. Next, we computed the mean proportion of these questions formulated with stereotype-consistent, stereotype-inconsistent, and stereotype-neutral topics.

### Results

#### Manipulation Checks

The *manipulation check of conversation partner age* showed that participants in the young conversation partner condition categorized their partner as belonging to the group of youngsters (*M* = 2.09, *SD* = 0.99) and estimated his age at *M* = 26.3 years, *SD* = 3.60, while participants in the old conversation partner condition categorized their partner as belonging to group of elderly (*M* = 6.32, *SD* = 1.02) and estimated his age at *M* = 69.9 years, *SD* = 7.17; *t*_group_(151) = 26.05, *p* < .001, *d* = 4.21; *t*_age_(149) = 47.70, *p* < .001, *d* = 7.77. We observed no differences between the three photographs used in both conditions.

The *manipulation check of activity stereotypicality* showed that stereotypically young-age activities were rated as significantly more expected for young people (*M* = 5.92, *SD* = 0.66) than old people (*M* = 2.29, *SD* = 0.97), *t*(152) = 33.08, *p* < .001, *d* = 2.67. By contrast, stereotypically old activities were more expected for old people (*M* = 5.81, *SD* = 0.82) than young people, *M* = 2.18, *SD* = 0.85, *t*(152) = 32.84, *p* < .001, *d* = 2.66. For stereotypically neutral activities, there was no difference in expectedness for young (*M* = 4.58, *SD* = 0.88) and old people (*M* = 4.37, *SD* = 0.86), *t*(152) = 1.92, *p* = .06, *d* = 0.16.

#### Hypothesis Testing

In line with Experiments 1 to 2, we confirmed *H1a* that people are likelier to address stereotype-consistent (vs. stereotype-inconsistent) conversation topics. An independent *t*-test showed no difference in the number of entered topics between the young and old conversation partner age conditions, *t*(151) = 1.70, *p* = .09, *d* = 0.3. However, participants in the young conversation partner condition rated their self-generated conversation topics as more stereotypical for a young person, compared with those in the old conversation partner condition, who rated their topics as more typical for an old person, *t*(151) = 9.17, *p* < .001, *d* = 1.48 (see Table 2).

**Table 2.** Means (and SDs) of Rated Conversation Topic Stereotypicality, and Rated Question Stereotypicality of Self-Formulated Questions About Stereotypically Young, Stereotypically Old, and Stereotypically Neutral Activities, as a Function of Conversation Partner Age (Young and Old; Experiment 3).

	Conversation partner age	
	Young (*n* = 79)	Old (*n* = 74)
Number of entered conversation topics	4.41^a^ (0.86)	4.14^a^ (1.10)
Rated conversation topic stereotypicality (young–old)	3.45^a^ (0.76)	4.66^b^ (0.88)
Mean number of entered questions		
About stereotypically young activities	2.71^a^ (0.89)	2.59^a^ (0.93)
About stereotypically old activities	2.44^a^ (0.88)	2.56^a^ (1.01)
About stereotypically neutral activities	2.75^a^ (0.87)	2.76^a^ (0.89)
Rated question stereotypicality for self-formulated questions . . .		
About stereotypically young activities	2.40^a^ (0.80)	3.80^b^ (1.45)
About stereotypically old activities	4.64^a^ (1.22)	5.72^b^ (0.79)
About stereotypically neutral activities	3.64^a^ (0.74)	4.24^b^ (0.65)

*Note. N* = 153. Rated topic/question stereotypicality 1 = *typical for/to young person*; 4 = *neutral*; 7 = *typical for/to an old person*. Means in rows with different subscript (^a, b^) are significantly different (*p* < .01 or lower) according to Bonferroni-adjusted pairwise comparisons.

Second, *H1b* stated that people prefer asking questions addressing stereotype-consistent (vs. stereotype-inconsistent) topics. In Experiment 3, participants formulated their own questions about stereotypically young/old/neutral activities and later rated these for question stereotypicality. A first test for *H2* is to look at the mean number of questions that participants generated about stereotype-consistent, stereotype-inconsistent, and stereotype-neutral activities. A mixed repeated-measures ANOVA with 2 (conversation partner age: young and old) × 3 mean number of entered questions (addressing stereotype-consistent activities, stereotype-inconsistent activities, and stereotype-neutral activities) with repeated measures on the last factor revealed a main effect of the number of questions entered, *F*(2, 302) = 19.08, *p* < .001, *η_p_*^2^ = .11. This revealed that the number of entered questions was higher for stereotype-consistent (vs. stereotype-inconsistent) activities (*p* = .006), confirming *H1b*. The mean number of entered questions for the neutral activities was significantly higher than both stereotype-consistent and stereotype-inconsistent activities (*p* < .006), but this may be due to the fact that there were less neutral activities.

A second test for *H1b* concerns looking at the self-rated question stereotypicality. We again conducted a 2 (conversation partner age: young and old) × 3 (rated question stereotypicality of questions addressing stereotypically young, stereotypically old, and stereotypically neutral activities) MANOVA, with repeated measures on the last factor. First, a main effect of question stereotypicality showed that formulated questions addressing stereotypically young activities were rated as more typically asked to a young person (*M* = 3.08, *SD* = 1.35), questions about stereotypically old activities are rated as more typically asked to an old person (*M* = 5.17, *SD* = 1.17), while stereotypically neutral activities are in between (*M* = 3.93, *SD* = 0.76), *F*(2,302) = 185.85, *p* < .001, *η_p_*^2^ = .55.

More relevant for *H1b* is a main effect of conversation partner age, *F*(1,151) = 109.97, *p* < .001, *η_p_*^2^ = .42, which showed that, irrespective of topic, participants in the young conversation partner condition rated their self-formulated questions as more typical for a young person (*M* = 3.56, *SE* = 0.07) compared with those in the old conversation partner condition (*M* = 4.59, *SE* = 0.07), confirming *H1b.*

Next, we focused on the coded question formulations. *H2* (focusing on frequency words) was tested in a similar way to Experiments 1 to 2. In line with Experiments 1 and 2, a main effect of the proportion of frequency adverbs showed that low-frequency adverbs (*M* = 0.14, *SE* = 0.01) were used more than high-frequency adverbs (*M* = 0.07, *SE* = 0.01), *F*(1,302) = 39.95, *p* < .001, *η_p_*^2^ = .21, which is understandable in a conversation with an unknown conversation partner.

Most relevant for *H2*, we observed an interaction between proportion of frequency adverbs and question topic, *F*(2,302) = 10.12, *p* < .001, *η_p_*^2^ = .06. This interaction showed that participants formulated fewer high-frequency questions when these addressed stereotype-inconsistent activities (vs. stereotype-consistent and stereotype-neutral activities). In contrast, participants formulate more low-frequency questions when these addressed stereotype-inconsistent activities (vs. stereotype-consistent and stereotype-neutral activities; see Table 3). Together, this replicates the results of Experiments 1 to 2 for self-formulated questions and again confirms *H2*. No other interaction effects were observed, *F* < 1.

**Table 3.** Means (and SD) of Expected Question Affirmation, Proportion of Valence Words, and Frequency Adverbs in Self-Formulated Questions About Stereotype-Consistent, Stereotype-Inconsistent, and Stereotype-Neutral Activities With Respect to the Age Category of the Conversation Partner (Experiment 3).

	Stereotype consistency of addressed activity		
	Consistent	Inconsistent	Neutral
Expected question affirmation	4.82^a^ (0.88)	3.14^b^ (1.06)	4.48^c^ (0.82)
Proportion of valence words in questions			
Positive valence	.20^a^ (.13)	.12^b^ (.11)	.16^c^ (.16)
Negative valence	.03^a^ (.07)	.03^a^ (.06)	.02^a^ (.05)
Proportion of frequency adverbs in questions			
High frequency	.08^a^ (.10)	.05^b^ (.07)	.09^a^ (.10)
Low frequency	.12^a^ (.13)	.16^b^ (.15)	.13^a b^ (.15)

*Note. N* = 153. Means in rows with different subscript (^a, b, c^) are significantly different (*p* < .01 or lower) according to Bonferroni-adjusted pairwise comparisons. Expected question affirmation scored on scale 1 = probably a negative answer (no); 4 = neutral; 7 = probably a positive answer (yes). Proportions are computed by dividing the number of self-formulated questions containing valence/frequency words by the total number of formulated questions addressing a specific activity.

For the use of valence words, we conducted a comparable MANOVA, but this time with proportion of valence words (positive and negative) as the repeated-measures factor. In line with Experiment 2, the use of positive words in self-formulated questions (*M* = 0.16, *SE* = 0.01) was more prevalent than the use of negative words (*M* = 0.02, *SE* = 0.00), *F*(1,302) = 283.77, *p* < .001, *η_p_*^2^ = .65. We also observed a main effect of question topic, *F*(1, 302) = 13.99, *p* < .001, *η_p_*^2^ = .09. Pairwise comparisons with Bonferroni corrections showed that, overall, valence words were used more in questions addressing stereotype-consistent topics (*M* = 0.11, *SE* = 0.01) vs. stereotype-inconsistent topics (*M* = 0.08, *SE* = 0.01, *p* < .001), or stereotype-neutral topics (*M* = 0.09, *SE* = 0.01, *p* < .02). Stereotype-inconsistent and stereotype-neutral topics did not significantly differ from each other (*p* = .07).

Most relevant for H3, we observed an interaction between proportion of valence words and question topic, *F*(2,302) = 14.15, *p* < .001, *η_p_*^2^ = .09. This interaction shows that participants formulated significantly more positive questions (e.g., “Do you *like* this TV show?” and “What do you *like* about Teletext?”) when these addressed stereotype-consistent activities (vs. stereotype-inconsistent and stereotype-neutral activities). Questions addressing stereotype-inconsistent topics contained significantly fewer positive questions than those addressing stereotype-neutral topics (confirming *H3a*). For the proportion of questions with negative words, no differences were observed, most likely because of the low number of questions containing negative words (see Table 3).

Finally, to explore reasons for adapting question formulations to stereotypes about one’s conversation partner, we measured the extent to which participants expected an affirmative (vs. denying) answer to the questions they formulated. The same MANOVA on *expected question affirmation* revealed a main effect of the repeated-measures factor, *F*(2, 302) = 149.79, *p* < .001, *η_p_*^2^ = .50, showing that participants expected a higher probability of affirmative answers for their questions formulated with stereotype-consistent (vs. stereotype-inconsistent or stereotype-neutral) activities. This effect could be partly due to the manipulated question topics, given that questions focusing on stereotype-consistent activities are more likely answered affirmatively. However, this result also suggests that participants formulate their questions toward affirmation.

### Discussion

Experiments 1 to 3 demonstrated how stereotypes associated with conversation partners’ social categories influence conversation topics and the types of questions asked.

Results supported *H1a*, in that participants indicated a higher preference for stereotype-consistent (vs. stereotype-inconsistent) conversation topics (Experiments 1 and 2) and rated the topics they spontaneously came up with themselves (Experiment 3) as stereotypical with respect to the conversation partner’s category. Similarly, and supporting *H1b*, participants indicated a higher preference for stereotype-consistent (vs. stereotype-inconsistent) question topics (Experiments 1 and 2) and rated their self-formulated questions as stereotypical (Experiment 3) with respect to their conversation partner’s social categories.

Likewise, participants preferred stereotype-consistent over stereotype-inconsistent question formulations. This was demonstrated for variations in question topic and for more subtle variations in question formulation related to frequency adverbs (Experiments 1–3) and question valence (Experiments 2–3). Regarding frequency adverbs (*H2*), participants indicated a stronger preference for - (Experiments 1 and 2), and self-formulated a higher proportion of - (Experiment 3), questions with high-frequency (vs. low-frequency) adverbs when these addressed stereotype-consistent (vs. stereotype-inconsistent) topics. For instance, when asking an old conversation partner about stereotype-consistent old activities, participants were likelier to prefer high-frequency adverb formulations (e.g., Do you *often* listen to classical music), while low-frequency adverb formulations were preferred more when addressing stereotype-inconsistent (vs. stereotype-consistent) activities (e.g., Have you *ever* played video games?).

With respect to question valence (*H3*), participants indicated a stronger preference for positively formulated questions when these addressed stereotype-consistent (vs. stereotype-inconsistent) topics, while they preferred negative formulations for questions addressing stereotype-inconsistent (vs. stereotype-consistent) topics (Experiment 2, not tested in Experiment 1). For instance, when asking a male conversation partner about stereotype-consistent male activities, participants were likelier to prefer positively formulated questions (e.g., Do you think Formula 1 is *interesting*?), while the preference for negative formulations (e.g., Do you think Formula 1 is *boring*?) decreased. Experiment 3 replicated this finding in self-formulated questions, but only for positive valence, as self-formulated questions with negative valence were rarely used in this study.

## Experiment 4

Experiment 4 aimed to explore the potential consequences of biased questions by focusing on recipient responses to questions reflecting stereotypes. We explore whether recipients detect that questions reflect stereotypes, and how this may affect perceptions of the conversation and partner. Participants imagined having a conversation and were presented with (and answered to) a number of questions posed by their conversation partner, which were either stereotypically consistent, inconsistent, or neutral with participants’ own gender. We measured various perceptions of the conversation and partner, perceived stereotyping, and gender stigma consciousness to test whether participants scoring high on this variable would be more sensitive to stereotype-confirming questions.

### Method

#### Participants and Design

Participants were recruited via snowball sampling and were offered a chance to win one of the two shop vouchers for €15 (approximately USD16). This resulted in a convenience sample of *N* = 256 Dutch voluntary participants who completed the online experimental questionnaire; 90 males (35.2%), 166 females (64.8%); *M*_age_: 31.0 years, *SD* = 13.5, minimum = 18, maximum = 68. Participants were randomly assigned to one of the three between-subjects conditions, in which the question sequence was stereotype-consistent, stereotype-inconsistent, or stereotype-neutral.

#### Procedure and Measures

Participants were informed that the study focused on what they deduced about a conversation partner from the questions she or he asked during a conversation. Participants were asked to imagine, to the best of their abilities, having a getting-acquainted conversation via online chat with a conversation partner about whom they knew little. No information or picture about this person was provided, and their gender was not revealed. Only the questions asked by the conversation partner during the conversation were shown. To facilitate imagining the conversation, participants were asked to answer to the questions (by typing in a text box) as they would answer them in real life.

Participants were then presented with 10 questions. The sequence always started with two neutral ice breaker questions, and then followed, depending on experimental condition, by eight questions asking about preferences for either stereotypically male, stereotypically female, or stereotypically neutral activities. In all conditions, questions were formulated similarly and with positive valence, and only varied between conditions in the mentioned activity (e.g., “Do you like rugby/ballet?”).

Next, participants were asked several questions about the conversation and their conversation partner, measuring the following variables (in presented order), and using 7-point Likert-type scales (1 = *completely disagree* to 7 = *completely agree*), unless mentioned otherwise. For all items, descriptives and Cronbach’s α, see Appendix Table A14 (https://osf.io/jc3fr/).

#### Dependent Variables

*Evaluation conversation* (two items, e.g., I thought this was a pleasant conversation). *Evaluation conversation partner* (three items, e.g., My conversation partner is friendly). *Perceived interest in person* (two items, e.g., My conversation partner really wanted to get to know me as an individual).

*Inferred gender conversation partner (*What do you think is the gender of your conversation partner? 1 = *probably male*; 4 = *neutral*; 7 = *probably female*). To improve comprehensibility, we recoded this value for male participants, such that the scale reflects 1 = *probably different as gender participant*; 4 = *neutral*; 7 = *probably same as gender participant*.

*Perceived gender stereotypes in conversation partner* measured the extent in which participants noted gender stereotypes and prejudice in their conversation partner (three items, e.g., My conversation partner has stereotypic expectations about my gender).

*Perceived stereotyping* measured the extent in which participants sensed to have been stereotyped based on their gender (five items, e.g., I felt stereotyped because of my gender).

*Stigma Consciousness* was included as a potential moderator variable and measured with the 10-item Stigma Consciousness Questionnaire (SCQ, Pinel, 1999).

##### Affirmative Answers

Next, participants were presented with their own answers to the eight questions that were part of the manipulation. For each answer, they indicated the extent to which they considered it a negating or affirmative answer, on a scale ranging from 1 = *completely negating*; 4 = *neutral*; 7 = *completely affirmative*. The eight responses were combined in a mean rating.

##### Manipulation Checks Question Topic

Finally, participants rated gender expectedness for all 24 stereotypically male, female, and gender-neutral activities that were used in the questions on 7-point scales ranging from 1 = completely a male activity through 7 = completely a female activity.

### Results

#### Manipulation Check and Correlations

A repeated-measures ANOVA on the rated gender stereotype expectedness showed that the manipulation was as intended. The eight activities addressed in the stereotypically male question sequence were rated as more typically male (*M* = 2.76, *SD* = 0.60), compared with the eight stereotypically female activities which were rated as more typically female (*M* = 5.16, *SD* = 0.55), while the eight neutral activities were in between (*M* = 3.99, *SD* = 0.17), *F*(2,508) = 1,031.240, *p* < .001, *η_p_*^2^ = .80. We observed no interaction, *F* < 1, or main effect, *F* (1,254) = 3.85, *p* = .051, *η_p_*^2^ = .015, of participant gender.

A correlation test on the dependent variables (see Appendix Table A15) revealed a number of interesting patterns. First, participants who perceived more stereotyping by their conversation partner evaluated both the imagined conversation (*r* = −.24, *p* < .001) and the conversation partner (*r* = –.19, *p* < .001) more negatively. In contrast, perceived interest in oneself as a person, which was negatively related to perceived stereotyping (*r* = −.18, *p* < .001), was associated with a more positive evaluation of the imagined conversation (*r* = .59, *p* < .001) and the conversation partner (*r* = .54, *p* < .001).

Second, participants who provided relatively more affirmative answers evaluated the imagined conversation more positively (*r* = .16, *p* < .001).

Third, the individual difference score in stigma consciousness was positively associated with perceived stereotyping by the conversation partner (*r* = .21, *p* < .001) and negatively related to giving affirmative answers (*r* = −.13, *p* < .05).

#### Effects of Stereotypicality of Question Sequence

We conducted a MANOVA with 3 (stereotypicality of question sequence: consistent, inconsistent, and neutral) × 2 (participant gender) on the dependent variables (see Table 4 for details).

**Table 4:** Means (and SDs) of Dependent Variables in Stereotypicality of Question Sequence Conditions Presenting Stereotype-Consistent, Stereotype-Inconsistent, and Stereotype-Neutral Questions With Respect to Participant Gender and MANOVA Statistics (Experiment 4).

	Stereotypicality of question sequence with respect to participant gender			Main effects						Interaction	
	Consistent (*n* = 93)	Inconsistent (*n* = 78)	Neutral (*n* = 85)	Stereotypicality of question sequence *df* = 2,250			Participant gender *df* = 1,250			*df* = 2,250	
Dependent variable				*F*	*p*	*η_p_* ^2^	*F*	*p*	*η_p_* ^2^	*F*	*p*
Evaluation conversation	4.21^a b^ (1.46)	3.78^b^ (1.44)	4.46^a^ (1.33)	3.60	**.03**	.03	5.34	**.02**	.02	1.25	ns
Evaluation conversation partner	4.67^a^ (0.98)	4.37^a^ (1.06)	4.73^a^ (0.90)	1.75	ns		1.76	ns		2.33	.1
Perceived interest in person	3.86^a^ (1.47)	3.27^b^ (1.46)	4.24^a^ (1.46)	7.90	**.000**	.06	5.39	**.02**	.02	<1	ns
Inferred gender conversation partner^1^	5.31^a^ (1.82)	1.62^b^ (0.98)	3.42^c^ (1.87)	90.29	**.000**	.42	1.06	ns		<1	ns
Perceived stereotypes in conversation partner	4.98^a^ (1.21)	3.26^b^ (1.07)	3.10^b^ (1.94)	67.70	**.000**	.35	11.23	**.001**	.04	<1	ns
Perceived stereotyping by conversation partner	3.45^a^ (1.26)	2.97^b^ (1.17)	2.13^c^ (1.05)	27.27	**.000**	.18	10.64	**.001**	.04	<1	ns
Affirmative answers	4.25^a b^ (1.38)	3.78^b^ (1.79)	4.74^a^ (1.11)	7.09	**.001**	.05	<1	ns		<1	ns

*Note. N* = 256 (^1^two missing values in inconsistent condition, *N* = 254). Means in rows with different subscript (^a, b, c^) are significantly different (*p* < .03 or lower) according to Bonferroni-adjusted pairwise comparisons.

Bold *p* values are significant (*p* < .05), ns = nonsignificant.

Inferred gender conversation partner was recoded, such that the scale reflects 1 = probably different as gender participant; 4 = neutral; 7 = probably same as gender participant.

Reported *F* statistics are univariate between-subjects effects from MANOVA including all dependent variables, except inferred gender conversation partner which was analyzed in a separate univariate ANOVA, because of missing values (*df* = 2,248 and 1,248).

Significant main effects of participant gender showed higher values for female than male participants on evaluation of conversation (*M*_female_ = 4.31, *SD* = 1.32; *M*_male_ = 3.89, *SD* = 1.58) and perceived interest in person (*M*_female_ = 3.96, *SD* = 1.52; *M*_male_ = 3.53, *SD* = 1.46), but higher values for male than female participants on perceived stereotypes in conversation partner (*M*_male_ = 4.10, *SD* = 1.37; *M*_female_ = 3.68, *SD* = 1.48) and perceived stereotyping by conversation partner (*M*_male_ = 3.14, *SD* = 1.32; *M*_female_ = 2.72, *SD* = 1.25).

First, main effects of stereotypicality of question sequence showed that participants confronted with stereotype-inconsistent questions about their own gender were more negative about the conversation, perceived the least interest in themselves as a person, and gave the least affirmative answers, compared with the consistent and/or neutral condition. Participants confronted with stereotype-consistent (vs. stereotype-inconsistent and stereotype-neutral) questions about their own gender, however, perceived more gender stereotypes and stereotyping by their conversation partner.

Interestingly, we also observed a main effect of stereotypicality of question sequence on inferred gender of the conversation partner, which shows that with inconsistent questions participants expect their conversation partner to be of different gender as themselves, while with consistent questions participants expect their conversation partner to be of the same gender. This is also in line with the significant positive correlations, showing that participants who perceived more stereotypes in, and stereotyping by, their conversation partner were more likely to infer their conversation partner was of the same gender (*r* = .38, *p* < .001; *r* = .13, *p* < .05, respectively). This suggests that participants infer that the question topic of their conversation partner may also be driven by this person’s own (stereotypically expected) interests, and that stereotype-consistent questions are most expected in same (as opposed to mixed) gender conversations. For instance, if a conversation partner asks questions about a stereotypically male activity to a female person, it is assumed that the conversation is likely male. The fact that we do not observe an interaction effect of participant gender on this recoded variable shows that this effect is consistent across male and female participants.

No other interactions were observed. Main effects of gender are reported in the note below Table 4.

### Discussion

A number of conclusions can be drawn about how questions reflecting stereotypes affect recipient responses. First, recipients appear to be able to detect that questions reflect a questioner’s stereotypes. That is, participants confronted with stereotype-consistent (vs. stereotype-inconsistent and stereotype-neutral) questions about their own gender perceived more gender stereotypes in, and stereotyping by, their conversation partner. Second, the perceived stereotypicality of questions appears to affect the evaluation of the conversation and partner.

On one hand, asking stereotype-consistent questions, that are expected to be answered affirmatively, can smoothen an interaction. In stereotype-inconsistent questions, fewer affirmative answers were provided than in the stereotype-consistent and stereotype-neutral conditions. On the other hand, the sense of being stereotyped (here based on one’s gender) has negative consequences. As evidenced by the pattern of correlations, people prefer to be judged as individuals. Thus, when it becomes too obvious that a conversation partner shows no interest in oneself as a person, but instead relies on generic gender stereotypes, this has negative consequences for the evaluation of the conversation and partner.

Finally, results on stigma consciousness show that individuals differ in sensitivity for stereotyping by a conversation partner. Individuals who score higher on stigma consciousness were more likely to perceive stereotyping by the conversation partner and were also less likely to go along by giving affirmative answers.

## General Discussion

Building on previous work on positive hypothesis testing (Nickerson, 1998; Sanbonmatsu et al., 1998; Trope & Liberman, 1996) and social information seeking (Dumont et al., 2003; Johnston, 1996; Swann & Giuliano, 1987), we tested whether and how stereotypes about a conversation partner influenced topic choice and question asking. Evidence of the few earlier studies focusing on the role of stereotypes in question-asking behavior was inconclusive (Dumont et al., 2003; Johnston, 1996). First, our findings, across well-powered Experiments 1 to 3, show a higher likelihood of addressing conversation topics consistent with stereotypic expectancies about conversation partners (which were merely manipulated by means of a picture). Likewise, participants preferred gender and age stereotype-consistent over stereotype-inconsistent question formulations.

Further extending these earlier studies (Dumont et al., 2003; Johnston, 1996), we demonstrated that these stereotypes are reflected not only in variations in question topics but also in more subtle variations in question formulations. When questions address stereotype-consistent topics, they are likelier formulated with high-frequency adverbs and with positive valence, while questions addressing stereotype-inconsistent topics tend to contain low-frequency adverbs and negative valence. Thus, when questioners address stereotype-inconsistent topics, the use of different valence or frequency adverbs in question formulations can reveal their stereotypic expectancies. In addition, results of Experiment 4 suggest that recipients are sensitive to detect that questions reflect stereotypes about themselves, which can influence the evaluation of the conversation and partner.

## Theoretical implications

Various processes may explain the tendency to formulate questions in line with activated stereotypic expectancies about one’s conversation partner. First, previous research observed an individual tendency of questioners to follow a positive test strategy to one’s initial hypotheses (Klayman & Ha, 1987; Nickerson, 1998; Trope & Liberman, 1996). As stereotype-consistent (vs. stereotype-inconsistent) activities are both expected to occur more frequently and to be more preferred, we assume that these stereotype-induced hypotheses are reflected in the use of frequency adverbs and valence words.

Second, in interactive conversations, positive hypothesis testing can follow for social and pragmatic reasons. It has been argued that stereotype-consistent questions are preferred because they are expected to trigger positive yes answers (Dumont et al., 2003) and thereby result in a more pleasant interactions (Holtgraves, 2001). Discourse analyses (Holtgraves, 2001) showed that, to render an interaction as smoothly and pleasantly as possible, conversation partners seek agreement and avoid uttering disagreement. Questioners can help to avoid such responses by asking stereotype-consistent questions that may be expected to be answered affirmatively. Moreover, introducing relevant topics is part of cooperative communication (Grice, 1975). This can thus be seen as socially adaptive, as a social skill (Dardenne & Leyens, 1995; Leyens et al., 1998; Snyder, 1992).

In line with this preference for agreement (Holtgraves, 2001), Experiment 4 revealed that the more affirmative answers recipients provided, the more positively they evaluated the imagined conversation. Importantly, however, sensing that one is being stereotyped by a conversation partner (as opposed to being judged as an individual) was related to a negative evaluation of the conversation and conversation partner. Thus, while relying on stereotypic expectancies allows people to adapt their conversation topics and questions to their conversation partner (Dragojevic et al., 2015), it may also turn to an excessive reliance on generalized stereotypic beliefs while an individual’s idiosyncratic characteristics are ignored. Particularly, recipients scoring high on stigma consciousness appear to be sensitive to this, while questioners may not always be aware that their stereotypes leak through.

Linguistic biases in communication toward members of minority groups (e.g., based on race, gender, and sexual orientation) have been described as microaggressions—subtle insults directed toward a person that threatens and demeans the target (Sue, 2010). While the people perpetrating them are usually unaware they are causing harm and often intend no offense, targets may be sensitive to the subtleties in language that reveal they are being categorized and associated with (negative) stereotypic traits. Being stereotyped—albeit by means of very subtle linguistic cues—may have several serious effects on targets. It may induce them to confirm expectancies conveyed by the speaker as a self-fulfilling prophecy (Hummert et al., 2004), can induce impaired performance as a result of stereotype threat (Shapiro & Neuberg, 2007), or improved performance as a result of stereotype lift (Walton & Cohen, 2003) can lower self-esteem (Bourguignon et al., 2015) and eventually even deteriorate mental and physical health (Pascoe & Richman, 2009). Thus, even though microaggressions are typically not classified as explicit cases of racism and/or prejudice, they can still have a profound negative impact on the people targeted.

An important question is whether biased question asking leads to stereotype confirmation and stereotype maintenance. It is often argued that positive hypothesis testing (i.e., one-sided information seeking) alone is insufficient to lead to confirmation because this depends on whether confirmative responses are provided (e.g., Capellini et al., 2017; Fiedler & Walther, 2003; Klayman & Ha, 1987; McKenzie, 2004). That is, questions based on an initial hypothesis may be answered by “yes” (which supports the hypothesis) or by “no” (which disconfirms it; Klayman & Ha, 1987; Leyens et al., 1998). Research on symmetric versus asymmetric social hypothesis testing argues that positive testing mainly leads to stereotype confirmation when the questions asked either vary in eliciting confirming or disconfirming evidence, or when the elicited answers do not have the same diagnosticity to confirm or disconfirm the tested hypothesis (Capellini et al., 2017). A question is asymmetric confirming when a provided confirming answer increases the hypothesis likelihood, but a disconfirming answer does not weaken it as much (Capellini et al., 2017). Research has found that when the tested social hypothesis is particularly salient and cognitively accessible (e.g., stereotypes), an asymmetric search strategy (more leading toward confirmative answers) tends to be preferred (Cameron & Trope, 2004; Capellini et al., 2017; Trope & Thompson, 1997).

It is important to note, however, that stereotype confirmation not only occurs as a result of one immediate question answer. It also results from factors occurring at a broader conversational level. First, the conversational topic choice can have an effect. For instance, by asking a question to a female conversation partner about the topic shopping, or child care, the conversation is directed toward these stereotypically female topics. This makes the conversation content in line with the stereotype. Second, the tendency to agree and go along with one’s conversation partner (Holtgraves, 2001; Zuckerman et al., 1995) pushes conversation partners toward answering affirmatively about the chosen topic. Third, even independent of the diagnosticity of the answers, positive hypothesis testing tends to result in a relatively higher amount of (confirmatory) learning input (e.g., linking women and child care), and this larger sample size of observations alone can result in its confirmation (Fiedler et al., 1999). Finally, questioners have a tendency toward interpreting answers in a stereotype-confirming manner. So, even when disconfirming answers are given, they may be perceived as less disconfirming when the target is fitting the stereotype (Cameron & Trope, 2004). Together, these effects of stereotypes on communicative behavior can lead to their confirmation and maintenance (Cameron & Trope, 2004; Dumont et al., 2003; Snyder & Stukas, 1999).

In general, studies on social hypothesis testing did not systematically focus on specific differences in question formulation, like we did in these studies. Future studies might focus on the extent in which the topic and question formulation differences we focused on direct immediate responses and the course of conversations as a whole, and result in stereotype confirmation.

## Strengths, Limitations, and Future Research

In our experiments, participants imagined having a getting-acquainted conversation, in which stereotypes were activated merely by presenting a photograph of an individual from a given social category. Although we think our scenario is more ecologically valid than the “testing a personality trait” approach that has been used in previous research, it is still limited in that it is not a real interaction. Future research could test the interactive dynamics of question asking in actual conversations and investigate when interviewees will follow the lead of stereotype-consistent questions and acquiesce by giving confirming answers, or instead give a disconfirming reply, and how such replies in turn affect the questioner’s follow-up questions and the course of the interaction. Interestingly, Johnston (1996) also looked at the effects of replies received in their bulletin board method and showed that participant tendency for stereotype-confirming information seeking increased after receiving stereotype-inconsistent information.

The reliance on stereotypes, and their reflection in question formulation, likely develops over the course of a conversation. Getting-acquainted conversations, with a completely unknown person, likely start with a reliance on generic stereotypes linked to the initial social categorization (e.g., age and gender), as this may be the only information available. When the conversation develops and more individualized knowledge about one’s partner is gained, more diagnostic questions may be asked that are focused on obtaining an accurate individualized impression (Leyens et al., 1998). It is highly relevant to study when and how such conversational dynamics result in an individualized impression or the confirmation of initial stereotypic expectancies in a speaker.

Conversation dynamics likely also depend on activated communication goals. Snyder (1992), for instance, distinguished between the “getting-to-know” and “getting-along” goals of the getting-acquainted situations. It appears that when people have a getting-to-know goal they are concerned with arriving at correct impressions and will thus attempt to look at people from a variety of perspectives and are open to new information. This likely reduces a reliance on generic stereotypes. Likewise, Johnston (1996) showed that stereotype-confirming information seeking decreased when accuracy and justification goals were made salient. Interestingly, Dumont et al. (2003) suggested that giving interviewers the goal to suppress stereotypes, for instance by means of forewarning or training, can be effective in reducing biased question asking.

Our experiments revealed that different subtle variations in question formulation reflect a questioner’s stereotypes about a conversation partner. There are likely other potential variations that we did not address. Some aspects of questions (i.e., question topic) reflect the content of an activated stereotype (e.g., old-age activities), which vary depending on the social category and activated stereotype of one’s conversation partner. Other linguistic aspects of questions relate to communicating unexpectedness (e.g., frequency words) or expected preference (e.g., valence words) and are thereby unrelated to the content of specific stereotypes at hand. Future research may address how stereotypes are reflected in (combinations of) other linguistic variations in question formulation, like for instance the use of negations (Beukeboom et al., 2010).

Our results have important implications. Questions influence recipient answers, and thereby the course, content, and outcome of conversations (Loftus, 1975; Semin & De Poot, 1997a, 1997b). By influencing question asking, stereotypes may thus play an important role in situations in which speakers interview conversation partners they do not know (e.g., job interviews and witness interrogations) and this, in turn, could lead to biased decision-making. In case of negative stereotypes and prejudice, people may thus unwittingly discriminate conversation partners by means of the content and formulation of the questions they ask.

## Notes

1. We observed no significant differences between the six photograph conditions in sex of participants, but the Photograph 2 (female) condition contained older participants compared with the other conditions (*p* = .02).

2. To explore whether age category perception would influence the effects on topic choice and question asking, we also measured *perceived entitativity* of both the age categories elderly and youngsters (see Online Appendix). As the variables did not show any correlations with the dependent variables we decided, for space considerations, not to report these analyses in the article.
